# Supplementary material for: A riboswitch gives rise to multi-generational phenotypic heterogeneity in an auxotrophic bacterium
Source: Nat Commun. 2020 Mar 5;11:1203. doi: 10.1038/s41467-020-15017-1 (PMC7058034; doi:10.1038/s41467-020-15017-1)
Supplement: Supplementary file 1 — Supplementary Information [file 41467_2020_15017_MOESM1_ESM.pdf]

Supplementary Information

A riboswitch gives rise to multi-generational phenotypic heterogeneity in an auxotrophic bacterium

Hernandez-Valdes *et al.*

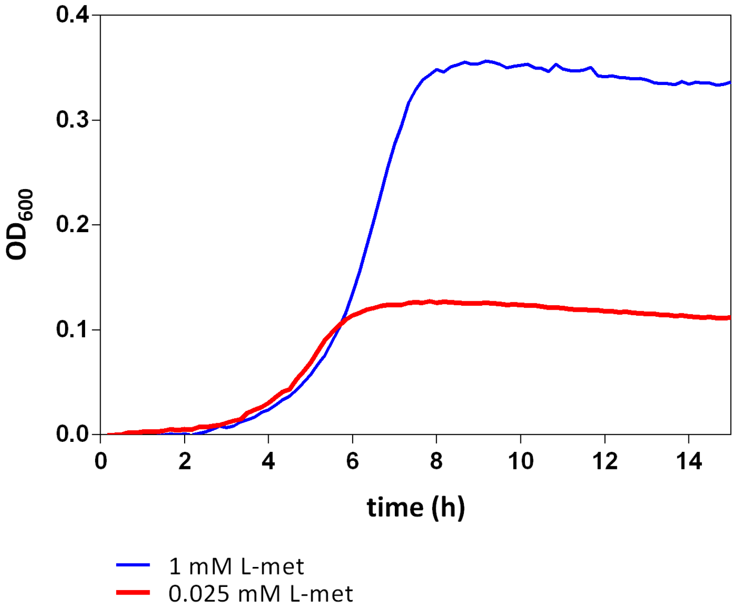

Supplementary Figure 1 | Methionine concentration is limiting for the growth of *L. lactis*. Growth curves (OD_{600}) of *L. lactis* MG1363 in CDM with low and high concentrations of methionine (0.025 mM in red and 1 mM in blue).

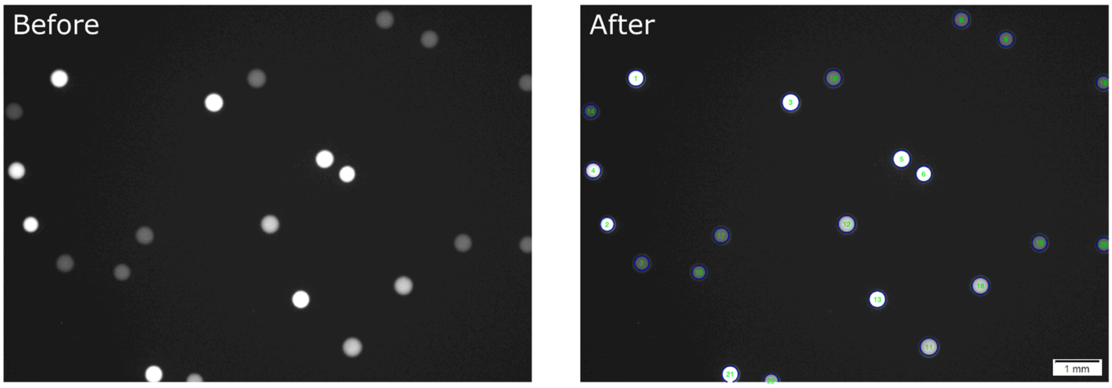

Supplementary Figure 2 | Example of automatic colony detection. *L. lactis* colonies on CDM-agar plate with low methionine concentration 0.025 mM show the two colony phenotypes (left image – before automatic detection), both phenotypes are efficiently detected (right-after detection). Scale bar, 1 mm.

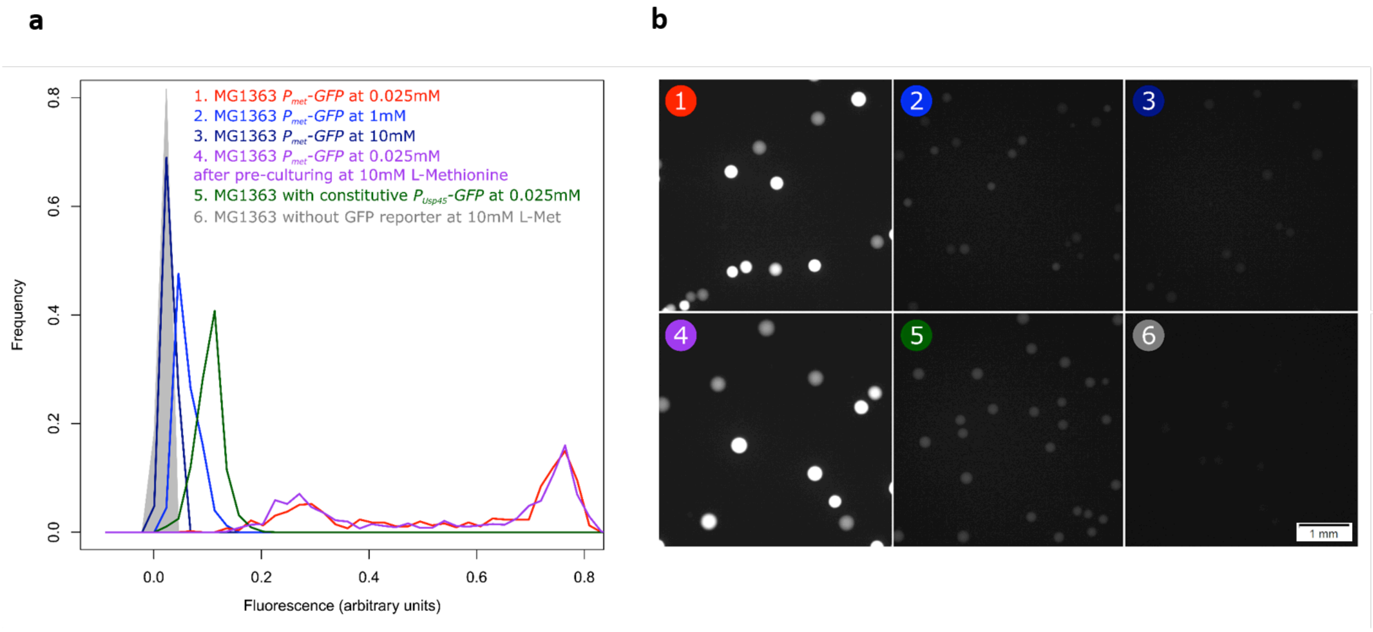

Supplementary Figure 3 | Control data in the colony analysis. a, Frequency of GFP+ and GFP- colonies using control strains and different growth conditions: 1-*Pmet-gfp* strain at low methionine concentration (0.025 mM, where the phenotypic heterogeneity is present), 2- *Pmet-gfp* strain at high methionine concentration (1 mM, no phenotypic heterogeneity, but low *met* expression), 3- *Pmet-gfp* strain at the highest methionine concentration (10mM, no phenotypic heterogeneity, and the lowest *met* expression), 4- *Pmet-gfp* strain at low methionine concentration (0.025 mM), but pre-cultured at high methionine concentration of 10 mM (phenotypic heterogeneity is present), 5- *Pusp45-gfp* strain at low methionine concentration (0.025 mM, homogenous expression of the constitutive *usp45* gene), 6- wild-type strain at high methionine concentration (10 mM), background fluorescence. **b**, examples of colonies on CDM-agar plate with the growth conditions tested in **a.** Scale bar, 1 mm.

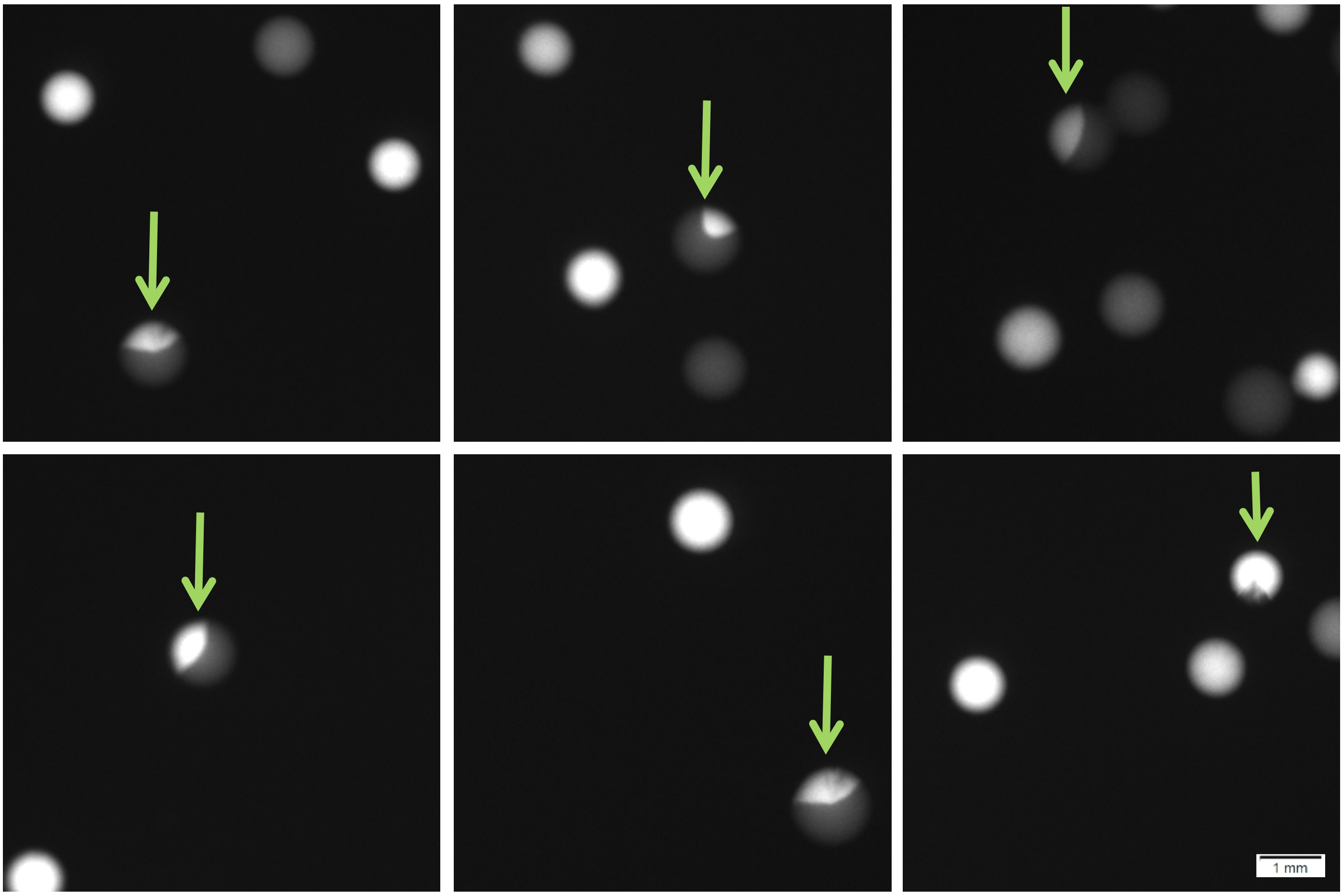

Supplementary Figure 4 | Examples of the rarely observed switching in *L. lactis Pmet-gfp* colonies. Colonies pointed by the green arrows show sectored and radial patterns in CDM-agar plates with low methionine concentrations (0.025 mM), inoculated with *L. lactis* cells and incubated for 48 h at 30 °C. Green fluorescence images are shown. Scale bar, 1 mm.

a

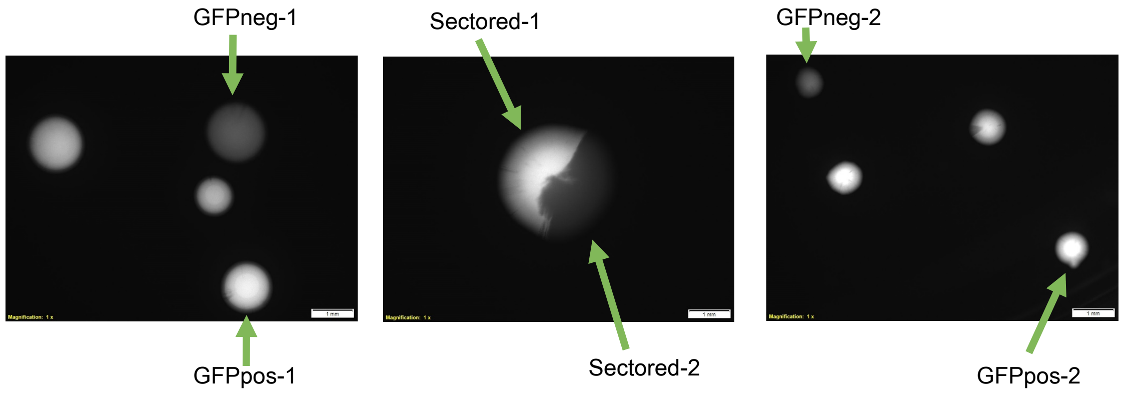

b

***met* promoter sequencing**		
Sample	*Phenotype*	*Percent Identitity**
GFPpos-1	GFP+	100%
GFPpos-2	GFP+	100%
GFPneg-1	GFP-	100%
GFPneg-2	GFP-	100%
Sectored-1	GFP+ (sectored)	100%
Sectored-2	GFP- (sectored)	100%

* Obtained by alignment of the DNA sequences to the met promoter reference sequence (*L. Lactis* subps. *cremoris* MG1363)

c

**Genomic DNA sequencing**			
Phenotype	*Annotation*	*Mapped reads**	*Predicted mutations*
GFP+	GFPpos-1	97.60%	not found
GFP-	GFPneg-1	99.00%	not found
GFP+ (sectored)	Sectored-1	98.30%	not found
GFP- (sectored)	Sectored-2	98.10%	not found

* Mapped reads using *Lactococcus lactis* subsp. *cremoris* MG1363, complete genome as reference.

Supplementary Figure 5 | DNA sequencing of different colony phenotypes of *L. lactis* *Pmet-gfp*. a, Colonies obtained in CDM-agar plates with low methionine concentrations (0.025 mM), inoculated with *L. lactis Pmet-gfp* cells and incubated for 48 h at 30 °C. Green arrows indicate the colony or sector of a colony taken to perform both, PCR colony and genomic DNA sequencing (see Methods). Green fluorescence images are shown. Scale bar, 1 mm. **b**, DNA sequencing of the *met* promoter, the percent identity value was obtained by alignment of the DNA sequences of the *met* promoter region in each colony (GFP+ indicated as GFPpos, GFP- indicated as GFPneg, and sectored colonies) by Clustal 2.1. The alignment was performed using the *met* promoter sequence as reference, and two samples per type of

colony. **c**, Identification of mutations in genomic DNA sequencing data obtained from samples of each colony phenotype. Breseq was used to map the sequencing reads to the reference genome AM406671 *Lactococcus lactis* subsp. *cremoris* MG1363, complete genome.

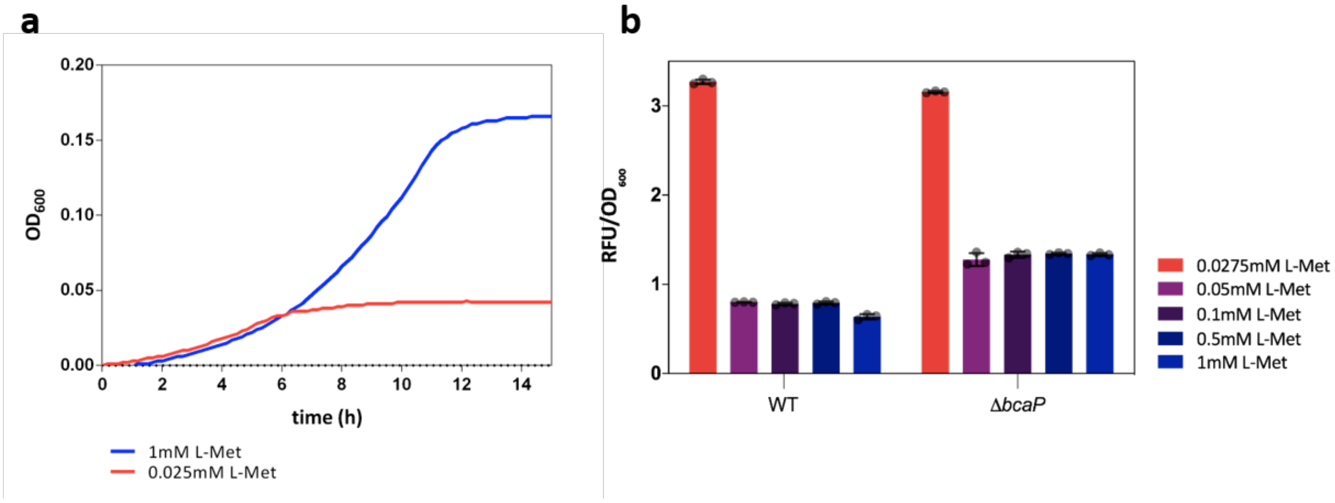

Supplementary Figure 6 | Effect of the deletion of *bcaP* from the chromosome of *L. lactis* on growth and *met* expression. **a**, The Δ*bcaP* strain grows slower compared to the wild type due to the fact that BcaP imports the branched-chain amino acids in *L. lactis*. Growth curves (OD_{600}) of *L. lactis* Δ*bcaP* in CDM with low and high concentrations of methionine (0.025 mM in red and 1 mM in blue). **b**, Population-level normalized GFP expression (RFU/OD_{600}) of *L. lactis* MG1363 cultures, wild-type (WT) and *bcaP* deletion mutant (Δ*bcaP*) strains, in CDM with increasing concentration of methionine (0.0275 mM to 1 mM, left to right bars). Dots represent independent experiments (n=3). Error bars represent standard deviation (SD) of the mean values of the three independent experiments.

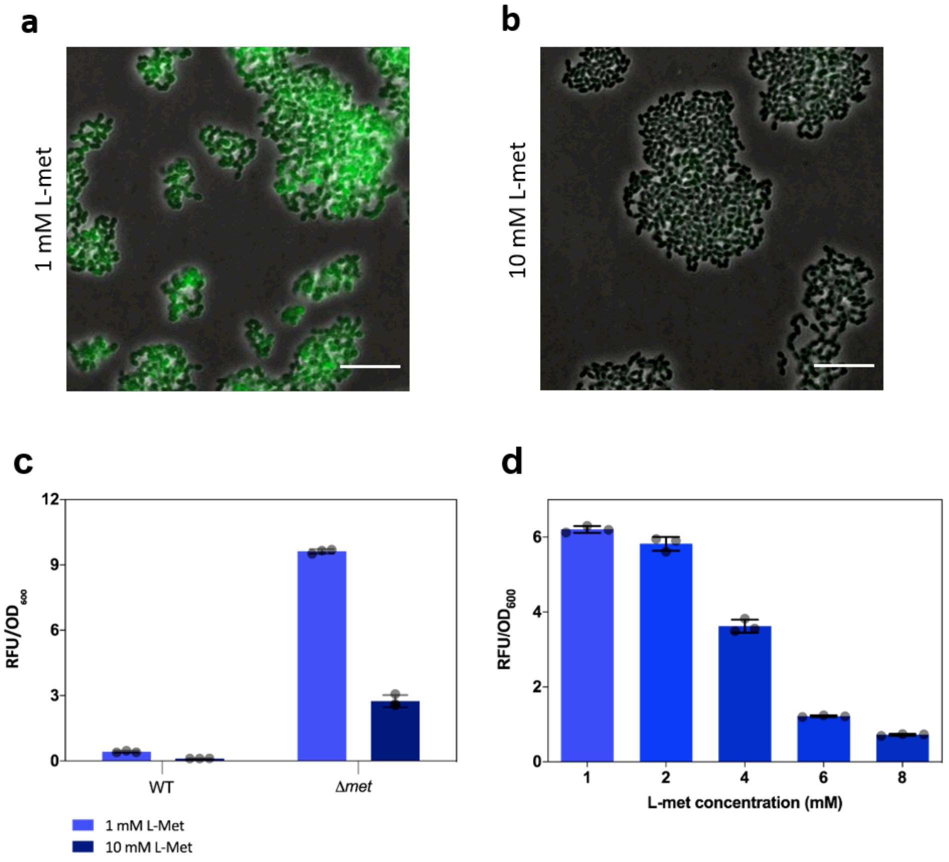

Supplementary Figure 7 | Deletion of the *met* promoter from the chromosome of *L. lactis* *Pmet-gfp* results in homogeneous activation of the *met* promoter. a and **b,** The *L. lactis* Δ*met Pmet-gfp* strain shows homogenous *met* expression. Snapshots of *L. lactis Pmet-gfp* cells by fluorescence microscopy at methionine concentrations of 1 mM and 10 mM. Images acquired by fluorescence microscopy. Overlays of green fluorescence and phase-contrast images are shown (Supplementary Video 5 and 6). Scale bars, 15 µm. **c***,* Since the *L. lactis* Δ*met* strain requires higher concentrations of methionine to grow, the *met* expression in this strain is activated at a methionine concentration of 1 mM. Population-level normalized GFP expression (RFU/OD_{600}) of *L. lactis Pmet-gfp* cultures, wild-type (WT) and *met* deletion mutant (Δ*met*) strains, in CDM with low and high concentrations of methionine (1 mM and 10 mM, left to right bars). Data are presented as mean ± S.D. Dots represent independent experiments (n=3). Error bars represent standard deviation (SD) of the mean values of the three independent experiments. **d**, The activation of the *met* expression in the *L. lactis* Δ*met* strain is inversely proportional to the methionine concentration. Population-level normalized GFP expression (RFU/OD_{600}) of *L. lactis* Δ*met Pmet-gfp* cultures in CDM with increasing concentrations of methionine (1 mM to 10 mM, left to right bars). Data are presented as mean ± S.D. Dots represent independent experiments (n=3). Error bars represent standard deviation (SD) of the mean values of the three independent experiments.

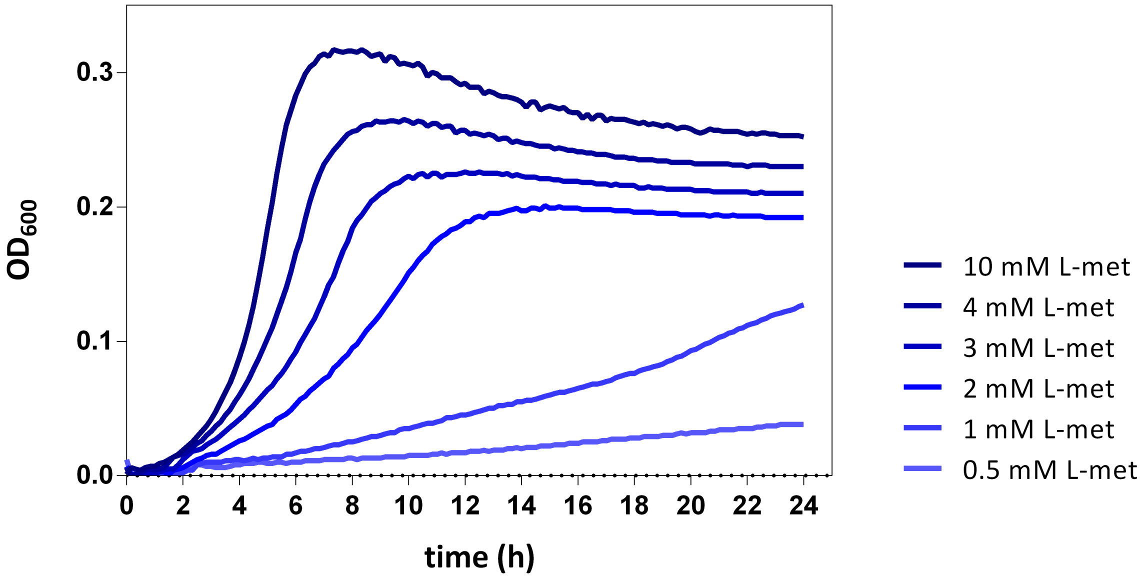

Supplementary Figure 8 | The *L. lactis* Δ*met* strain requires higher methionine concentrations to grow. Growth curves (OD_{600}) of *L. lactis* Δ*met* cultures, performed in CDM with different methionine concentrations (0.5 mM to 10 mM).

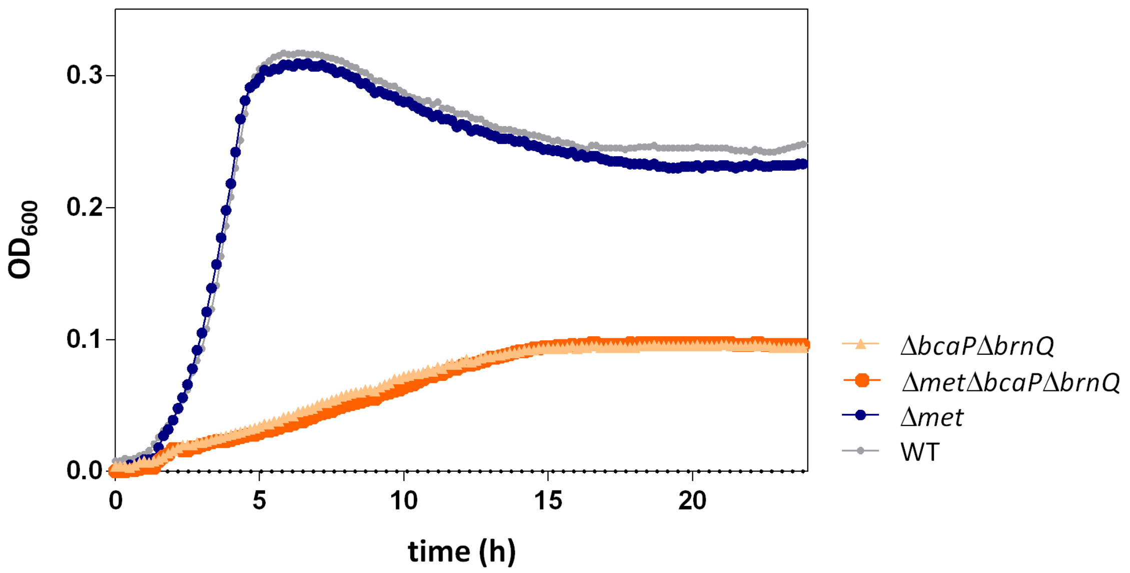

Supplementary Figure 9 | The BcaP and Met-transporters are the only two systems able to import methionine in *L. lactis*. Previous studies show that the lack of BcaP is partially compensated by a low-affinity BCAA transport system II carrier protein (BrnQ). Therefore, a triple deletion mutant strain (Δ*met*Δ*bcaP*Δ*brnQ)* was constructed in order to investigate whether another methionine transporter, besides Met and BcaP, is present in *L. lactis*. At a high methionine concentration of 10 mM, *L. lactis* Δ*met* (in dark blue) shows similar grow to the wild-type strain (gray) due to the methionine import via BcaP. In contrast, no differences in growth between the triple-mutant (Δ*met*Δ*bcaP*Δ*brnQ) (*in dark orange) and a double mutant (Δ*bcaP*Δ*brnQ)* (in light orange) were observed in CDM with a high methionine concentration of 10 mM. Thus, it appears that BrnQ is unable to transporter methionine. Although, the triple-mutant strain (Δ*met*Δ*bcaP*Δ*brnQ)* does grow in CDM with a prolonged lag phase (in dark orange) and reaches lower final cell-density compared to the wild-type strain (in gray).

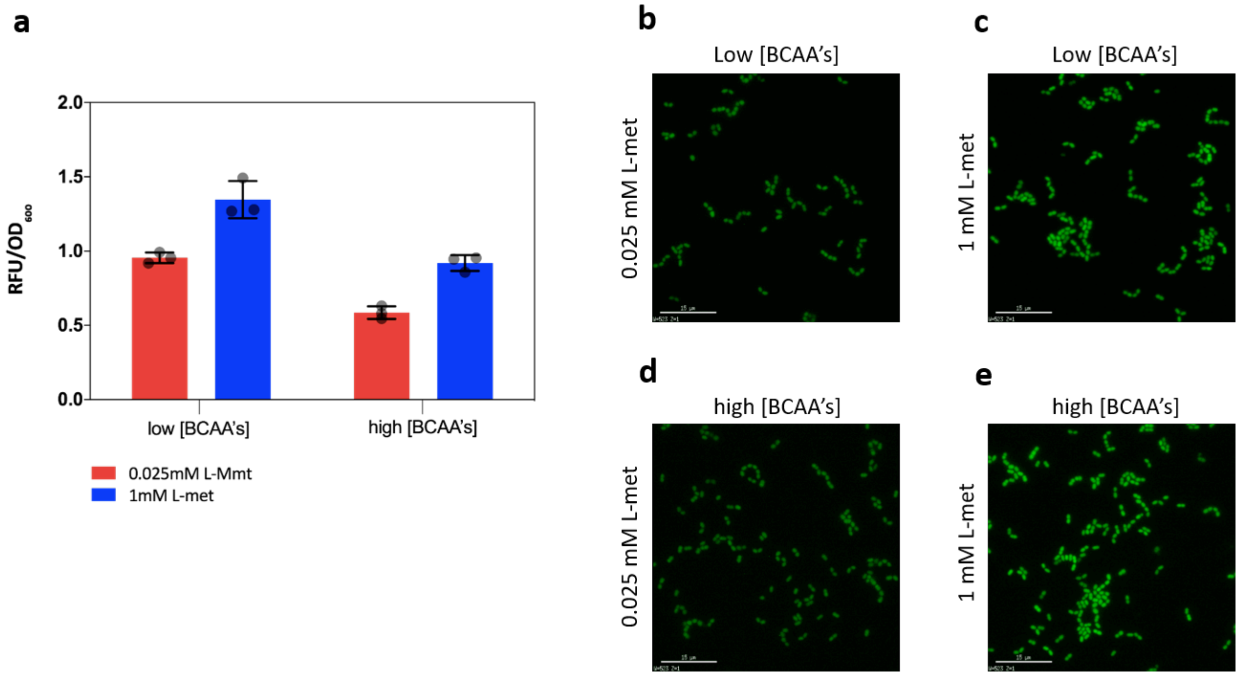

Supplementary Figure 10 | Methionine competes with branched-chain amino acids for BcaP. **a**, Population-level normalized GFP expression (RFU/OD_{600}) of *L. lactis* MG1363 *PbcaP-gfp* construct, in CDM with increasing concentration of methionine (0.025 mM to 1 mM, red and blue bars respectively), and the presence of low or high branched-chain amino acid concentration: low BCAA's (leucine 0.4 mM, isoleucine 0.2 mM, valine 0.23 mM) and high BCAA's (leucine 4 mM , isoleucine 2 mM and valine 2.3 mM). Data are presented as mean ± S.D. Dots represent independent experiments (n=3). Error bars represent standard deviation (SD) of the mean values of the three independent experiments. **b** and **c**, Snapshots of observations by fluorescence microscopy of *L. lactis PbcaP-gfp* at exponential growth phase in medium with low and high methionine concentrations (0.025 mM and 1 mM **b** and **c** respectively), performed in CDM with low branched-chain amino acids concentrations. **d** and **e**, Snapshots of observations by fluorescence microscopy of *L. lactis PbcaP-gfp* at exponential growth phase in medium with low and high methionine concentrations (0.025 mM and 1 mM, **d** and **e** respectively), performed in CDM with high branched-chain amino acids concentrations.

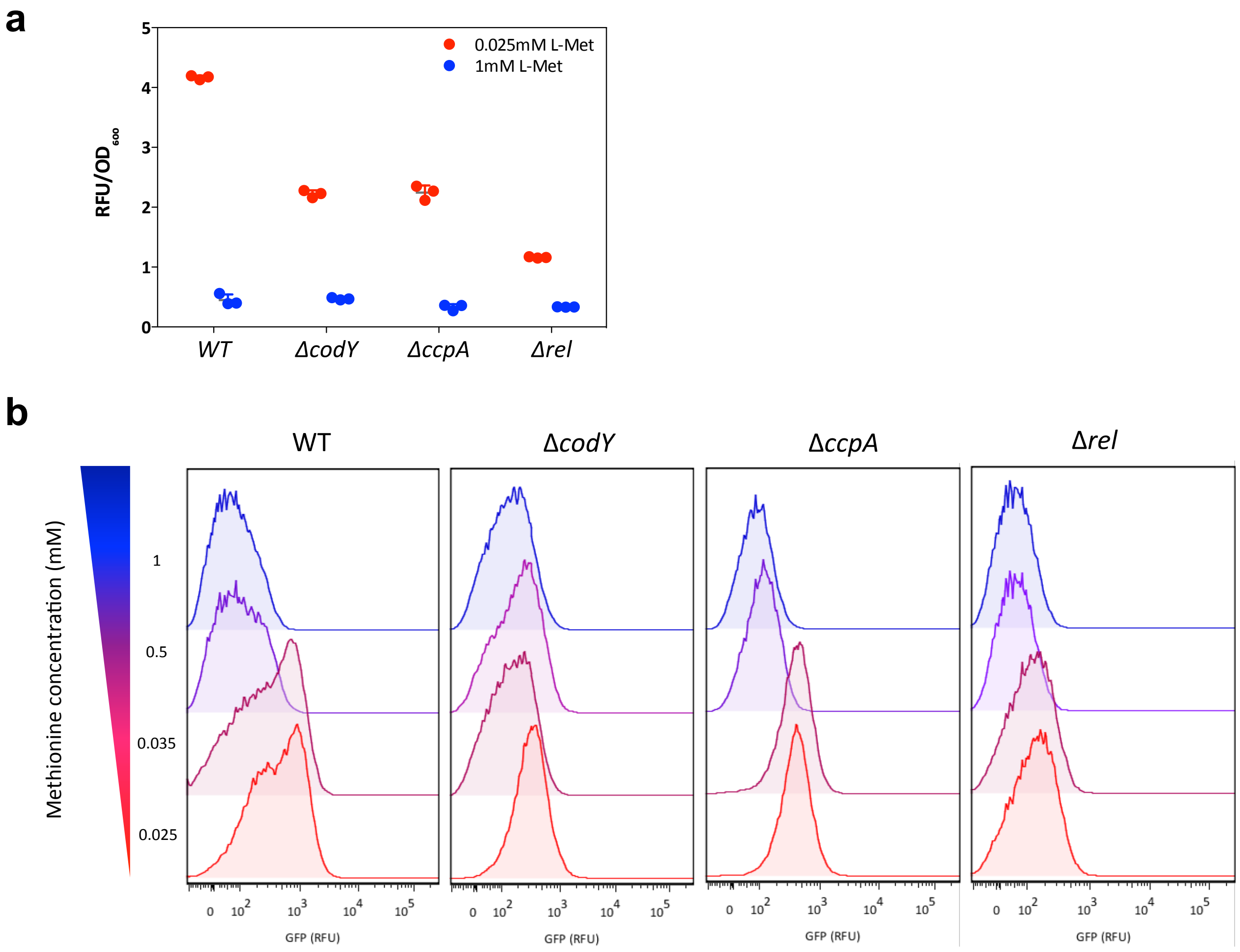

Supplementary Figure 11 | The role of global regulators on the expression of the *met* operon. **a**, Deletion of *codY, ccpA,* or *rel* from the chromosome of *L. lactis Pmet-gfp* decreases the activation of the *met* promoter at low methionine concentrations. Remarkably the deletion of *rel* shows the lowest *met* expression at low concentrations of methionine (0.025 mM). Furthermore, there is a similar effect with the deletion of *codY*, which may be explained by the fact that *bcaP* becomes highly expressed without the repression by CodY, and therefore the requirement of the Met transporter decreases. Note: the wild type shown in this figure is the same as in Figure 6. Population-level normalized GFP expression (RFU/OD_{600}) of *L. lactis Pmet-gfp* cultures in CDM with low (red) and high (blue) concentrations of methionine (0.025 mM and 1 mM). Dots represent each independent experiments (n=3). Error bars represent standard deviation (SD) of the mean values of the three independent experiments. **b**, Single-cell GFP measurements by flow cytometry at different concentrations of methionine (0.025 mM to 1 mM). 10,000 ungated events for each sample are shown.

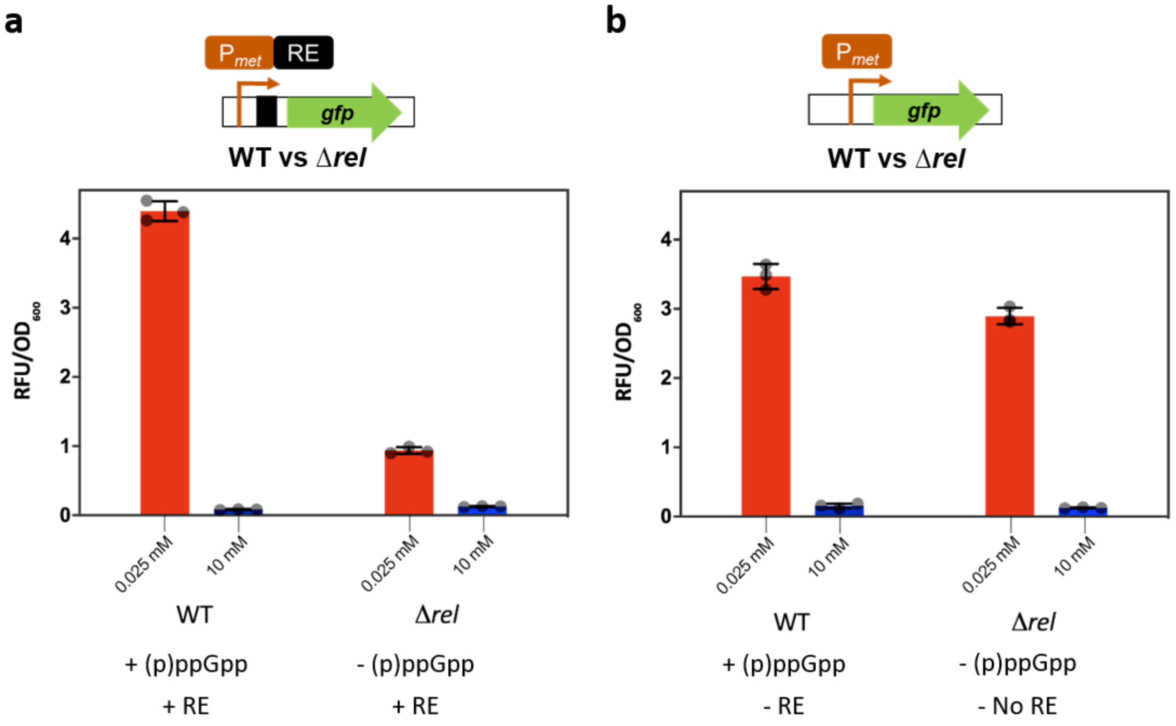

Supplementary Figure 12 | Rel plays a role in the regulation of the *met* promoter. a, Population-level normalized GFP expression (RFU/OD_{600}) of *L. lactis* cultures wild-type strain and *rel* deletion mutant, bearing the *met* promoter (including the regulatory element RE) fused to the *gfp* gene. Cells are propagated in CDM with low and high methionine concentrations (0.025 mM in red and 10 mM in blue). Data are presented as mean ± S.D. Dots represent independent experiments (n=3). Error bars represent standard deviation (SD) of the mean values of the three independent experiments. **b**, Population-level normalized GFP expression (RFU/OD_{600}) of *L. lactis* cultures wild-type and *rel* deletion mutant strains, bearing a variant of the *met* promoter (lacking the regulatory element RE) fused to the *gfp* gene. Cells are propagated in CDM with low and high methionine concentrations (0.025 mM in red and 10 mM in blue). Error bars represent standard deviation (SD) of the mean values of three samples (n = 3).

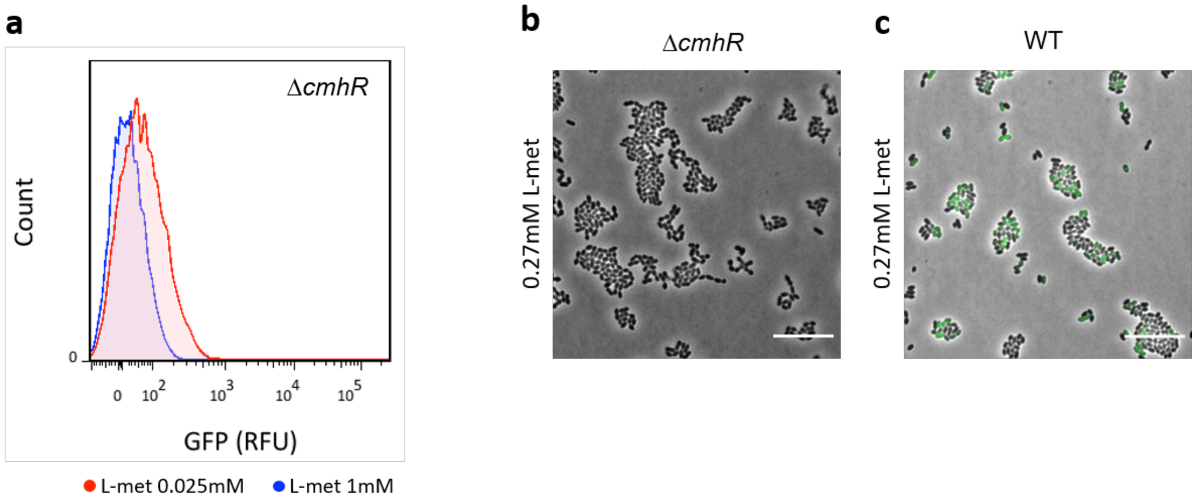

Supplementary Figure 13 | The transcription factor CmhR regulates the *met* promoter. **a,** The deletion of *cmhR* from the chromosome of *L. lactis Pmet-gfp* results in no *met* expression at different methionine concentrations (0.025 mM in red, and 1 mM in blue). 10,000 ungated events for each sample are shown. **b** and **c** Snapshots of cells of the wild-type and Δ*cmhR* strains by fluorescence microscopy in standard CDM that contains 0.27 mM methionine. No Met-transporter expression is observed in the Δ*cmhR* strain. Overlays of green-fluorescence and phase-contrast images are shown. Scale bars, 15 µm.

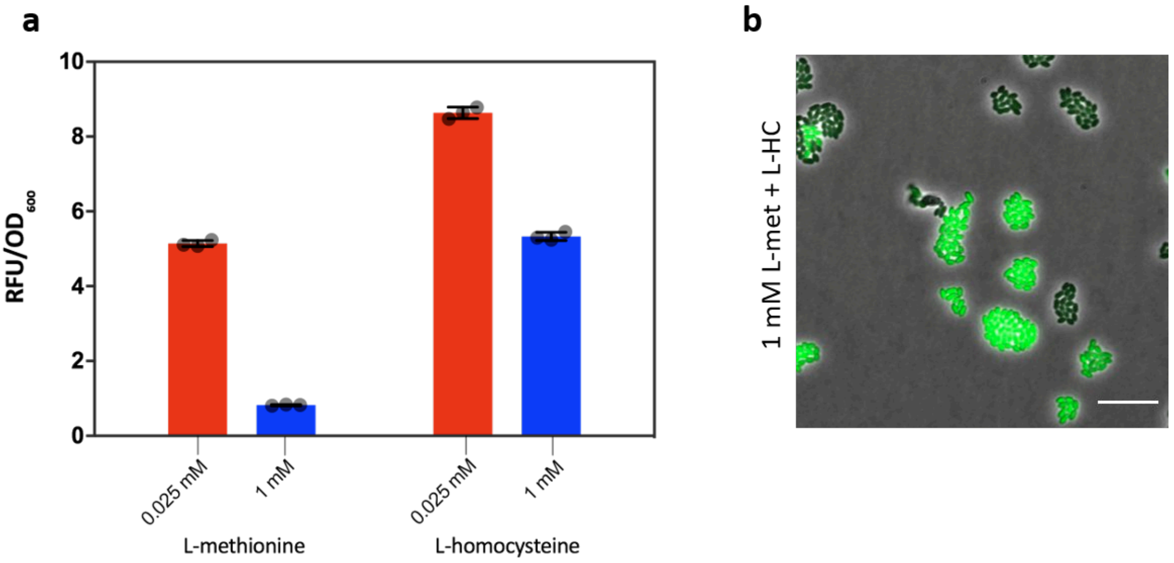

Supplementary Figure 14 | L-homocysteine increases the expression of the Met-transporter. **a**, Since homocysteine can be converted to methionine, *L. lactis* is able to grow in CDM containing L-homocysteine instead of methionine. The expression of the *met* operon is enhanced with the presence of L-homocysteine, even at a high concentration of methionine (1mM). Population-level normalized GFP expression (RFU/OD_{600}) of *L. lactis Pmet-gfp* cultures, in CDM with low and high methionine concentration (0.025mM and 1mM) and high and low L-homocysteine concentration (0.025 mM and 1 mM). Data are presented as mean ± S.D. Dots represent independent experiments (n=3). Error bars represent standard deviation (SD) of the mean values of the three independent experiments. **b** Snapshot of microscopy observation of *L. lactis Pmet-gfp* strain in CDM with high methionine (1 mM) and L-homocysteine at a concentration of 0.27 mM (Supplementary Video 8). An overlay of green fluorescence and phase-contrast images is shown. Error bars represent standard deviation (SD) of the mean values of three samples (n = 3). Scale bar, 15 µm.

Name: T-box
Type: RNA regulatory element
Effector: Met-tRNA

Regulog T-box(Met) *RegPrecise 3.0 database*

Regulated operon	Function	Genomes
metNPQ	Methionine ABC transporter	*L. lactis* subsp. *lactis* IL1403, *L. lactis* subsp. *cremoris* SK11,

L. lactis **subsp.** ***cremoris*** **MG1363** *RibEx: Riboswitch explorer*

	T-box motifs	Putative regulated operon	Function	
Motif 1	cttaaATCAAGAATGGTACCACGATATAGCTCGTTtcttt	*plpABCD-ydcB-ydcC*	Methionine ABC transporter	P-value 3.38e-08
Motif 2	tttatGAAATGGGCTCTTTTTGTTTAGTCAGAttgtt			
Motif 3	tcagtACAGAGAGCTTGTAGCGGTGAgagca			

Supplementary Figure 15 | A T-box riboswitch is located upstream the *met* operon. The T-box riboswitch is a well-known regulatory element (RFAM: RF00230), which is predicted by RegPrecise 3.0 database to regulate the *metNPQ* operon (named *met* operon in our study) in *Lactococcus lactis*. We identified three motifs belonging to the T-box leader in the genome of *L. lactis* MG1363 by RibEx: Riboswitch explorer, these motifs are located upstream the *met* operon (*plpABCD-ydcB-ydcC*) encoding the methionine ABC transporter, in a similar way to other species of *L. lactis*. The *P*-value is calculated by RibEx assuming a hyper-geometrical distribution, for each motif to be overrepresented in a given COG or KEGG pathway, with the Motif Alignment and Search Tool (MAST) algorithm[1,2].

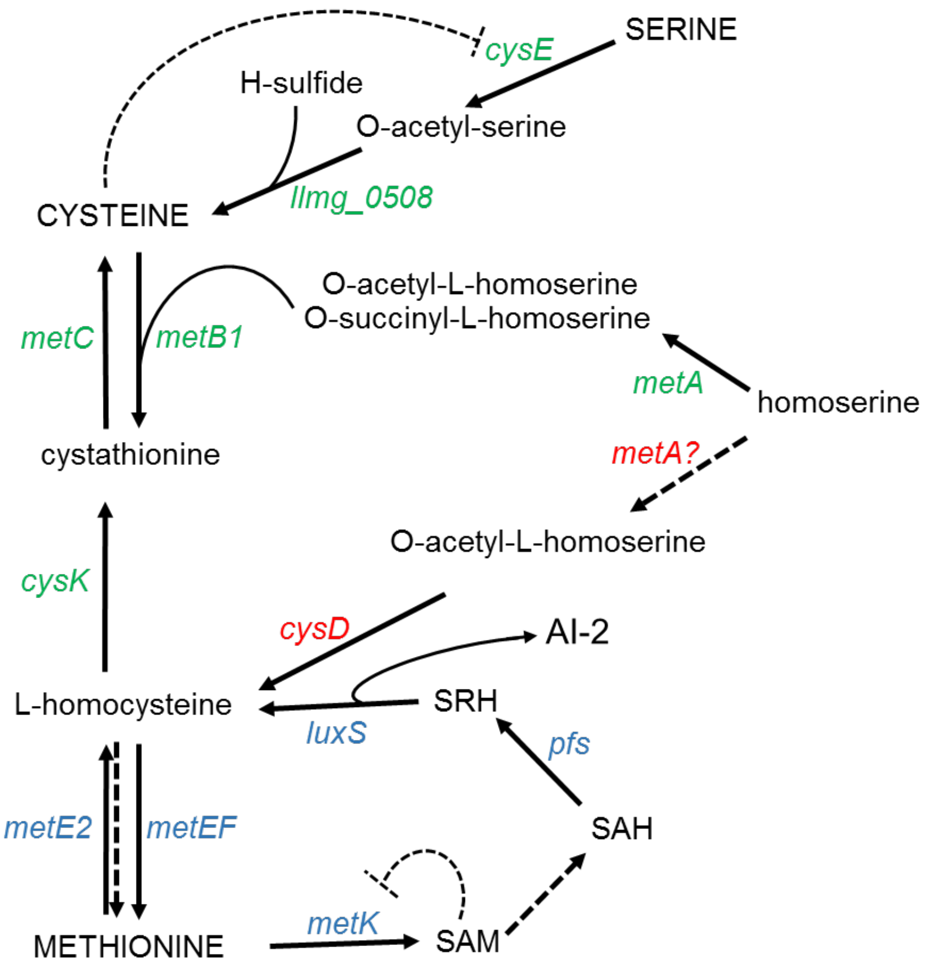

Supplementary Figure 16 | Impaired methionine biosynthesis pathways in *L. lactis* MG1363. Although methionine can potentially be synthesized using L-homocysteine as a substrate, experimentally this bacterium is an auxotroph for methionine. The pool of homocysteine is derived from a recycling pathway via *metk, pfs* and *luxS*, or via interconversion of homoserine into L-homocysteine by *metA* and *cysD*. We speculate that either the amounts of methionine produced by this pathways is not enough to let the bacteria proliferate or the biosynthetic pathways are impaired by gene mutations. In a review of the literature, Sperandio *et al*, 2010 described the sulfur amino acid metabolism in the *L. lactis* IL1403 strain[3], and showed that cysteine might enter by an interconversion pathway to methionine. This conversion is not possible in *L. lactis* MG1363 due to the lack of the enzyme YtjE that converts cystathionine into homocysteine. In this diagram, we highlight three transcriptional regulators participating in these biosynthetic pathways: genes regulated by CmbR in green, genes regulated by CodY in red, and genes that are potentially regulated by CmhR in blue.

a **DNA**

In **red** regulatory element (RE)

5′AAAAAAAACTCCTTTTTGATGCGCACAAAAGGAGGGTTAGAAATTAGACGATTGTCTTAACACATCCCT

ACGCTGGCATTACCCAGATCAGGTCAGATGGTATTTCTCGCTACTGACAGACTTGTCAATAGACCCATTGT

GCAAATATTTATTTTTACAAGTTTAAGTTTAGCATAATTTATGTTGAAAAGCATTGATGGGAGATTAGAAT

TCTATTTTATAAATTAAACTTAAAAATTTTTTAAGGCAACATAAAAAATCAATAATCCAAAAAATTTGAAA

GTCTGTTAGAATAAATCTATACACAAATTCGGACAAAATAAAAGCGGTCGAGTAAGTAATTGTGAAAGTCA
+1

GTACAGAGAGCTTGTAGCGGTGAGAGCAAGCCTGAATGAAGCAATGAAAATGGCTTGATAAGCGTAGCAAA

AGTGAGGTTTTACCAAGCTTTTGACGGCATGGTTCTCGTTATTGAACTTAAAAGTTTGCTTTTGTAAAGTT

TATCTGAAAATTTTCAGTGAGCTACAAAGTGCTTAAATCAAGAATGGTACCACGATATAGCTCGTTTCTTT

TTATGAAATGGGCTCTTTTTGTTTAGTCAGATTGTTTTTGTTTGACGAATTTGTTTAGAGTCAGAAAATTG

GCAGGTGCACGGTTAATTTTCTGATATGTCGAGTACTGATTAACTAATAAGGAGGACAAAC**ATG**3′

b **RNA**

In **red** highly conserved nucleotides, **blue** terminator sequence, **green** the specifier for methionine

5′AUACACAAAUUCGGACAAAAUAAAAGCGGUCGAGUAAGUAAUUGUGAAAGUCAGUACAGAGAGCUUGUAGC
GA motif S-turn AG bulge

5'-CGGUGA-3'
5'-CUUUGA-3' ❶

GGUGAGAGCAAGCCUGAAUGAAGCAAUGAAA**AUG**GCUUGAUAAGCGUAGCAAAAGUGAGGUUUUACCAAGCUU
Stem I Specifier loop (Met)

UUGACGGCAUGGUUCUCGUUAUUGAACUUAAAAGUUUGCUUUUGUAAAGUUUAUCUGAAAAUUUUCAGUGAGC
Stem II

❷ 5'-AUGGUA-3'
5'-A----A-3'

5'-UCGUUU-3'
5'-UUUCUU-3' ❸

UACAAAGUGCUUAAAUCAAGAA**UGGU**ACCACGAUAUAGCUCGUUUCUUUUUAUGAAAUGGGCUCUUUUUGUUU
T-box

Δ60 bp ❹

AGUCAGAUUGUUUUUGUUUGACGAAUUUGUUUAGAGUCAGAAAAUUGGCAGGUGCACGGUUAAUUUUCUGAUA
Terminator

UGUCGAGUACUGAUUAACUAAUAAGGAGGACAAAC**AUG**3´

Supplementary Figure 17 | Analysis of the 5' UTR of the *met* operon. a, The DNA sequence shows the transcription start site (+1), and the regulatory element (RE) deleted in the P*met(-RE)-gfp* strain (Fig. 5d) is shown in red. **b**, The RNA sequence shows the identified domains, elements, and conserved nucleotides among T-box riboswitches. The identification of these structural elements is based on previous studies[4–6]. Moreover, the four mutations introduced to the riboswitch in this study (Fig. 6) are highlighted with numbers.

a **qRT-PCR primers design**

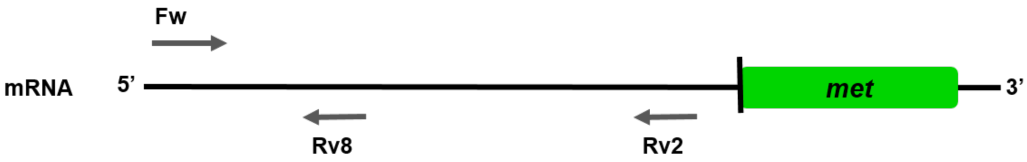

b **qRT-PCR results**

Set of primers: Fw + Rv2

	LOW MET		HIGH MET	
Strain	*met* Norm. to *rarA*	*met* Norm. to *rpoE*	*met* Norm. to *rarA*	*met* Norm. to *rpoE*
	0.025mM	***0.025mM***	***10mM***	***10mM***
WT	1.805501	2.528403	0.000048	0.000054
Δ*bcap*	3.326575	11.493275	0.000030	0.000083

Set of primers: Fw + Rv8

Strain	*met* Norm. to *rarA*	*met* Norm. to *rpoE*	*met* Norm. to *rarA*	*met* Norm. to *rpoE*
	0.025mM	***0.025mM***	***10mM***	***10mM***
WT	1.163251	1.629003	0.001582	0.001785
Δ*bcap*	3.425628	11.835501	0.000529	0.001481

CmhR activates *met* promoter: **Transcription ON**

CmhR does not activate *met* promoter: **Transcription OFF**

Supplementary Figure 18 | Quantitative analysis of the *met* expression. a, qRT-PCR was performed with two sets of primers: Fw+Rv2 and Fw+Rv8, based on the distance to the transcription start site. **b,** *met* expression at low (0.025 mM; red) and high (10 mM; blue) methionine concentrations, in the wild-type and *bcap* deletion strain. The results are normalized to the expression of *rpoE (*encoding for the DNA-directed RNA polymerase subunit delta) and *rarA* (encoding for a recombinase) genes. The expression of the *met* operon occurs at low methionine concentrations, whereas at high methionine concentrations the *met* expression is very low. Moreover, the deletion of *bcaP* causes higher *met* expression levels, since lacking the BcaP makes the Met transporter the only path left to import methionine. These results represent the mean values of three samples (n = 3).

Supplementary Table 1 | Oligonucleotides used in this study

Name	Sequence
metFw	5′ CTCTGCGCATGCTTTGGAATGAGGCTGATGATGAAGG 3′
metRv	5′ CTCGAGTTGATTTATTTTTCAAAATCAATTATTCCCCTTTG 3′
met(-RE)_Rv	5′ CTTTCACTCGAGCTTACTCGACCGCTTTTATTTTG 3′
bcaP_Fw	5′ TAAGAAGCATGCCAGTTGAAAGTTTTCACGAGGTTC 3′
bcaP_Rv	5′ TAAATCCTCGAGTTTCTGAATTTGTAACTAAATAATTTC 3′
A_metKO_Fw	5′ TTATTAGGTACCACTATGGATAAAAAGTTTAG 3′
A_metKO_Rv	5′ TTGACAAGAATTCTAAAAATAAATATTTGCACAATG 3′
B_metKO_Fw	5′ GGGAGAGGATCCGAATCCAAGAAACAGAAATAT 3′
B_metKO_Rv	5′ CACTATCTTTGTTTACAGGTTCGCGGCCGCTAGACTC 3′
met_promoter_Fw	5′ AAGCTGAGAAGAAGGATAGGGATA 3′
met_promoter_Rv	5′ GCAGCGATAGCAACAATTACAACAAC 3′
Fw (qRT-PCR)	5′ ACACAAATTCGGACAAAATAAAAG 3′
Rv2 (qRT-PCR)	5′ GAAAATTAACCGTGCACCTG 3′
Rv8 (qRT-PCR)	5′ CTTCATTCAGGCTTGCTCTC 3′
rpoE-Fw	5′ GAAGTCATCGCTCTTGACGAA 3′
rpoE-Rv	5′ TCTGGGTCGATTTCGTCTTC 3′
rarA-Fw	5′TGCAGAGCAGACAGCAAATC 3′
rarA-Rv	5′ ACACCGCGTTACCCTAGATG 3′
Mut1-Fw	5′GTCAGTACAGAGAGCTTGTAGCTTTGAGAGCAAGC 3′
Mut1-Rv	5′ GCTTCATTCAGGCTTGCTCTCAAAGCTACAAGCTC 3′
Mut2-Fw	5′ GCTACAAAGTGCTTAAATCAAGAAACCACGATATAGC 3′
Mut2-Rv	5′ GAAACGAGCTATATCGTGGATTCTTGATTTAAGC 3′
Mut3-Fw	5′ GGTACCACGATATAGCTTTCTTCTTTTTATGAAATGGGC 3′
Mut3-Rv	5′ CAAAAAGAGCCCATTTCATAAAAAGAAGAAAGCTATATCG 3′
Mut4-Rv	5′ CAAATTCCTCGAGCAAAAACAATCTGACTAAACAAAAAGAG 3′

Supplementary Table 2 | Constructed strains in this study

Name	Description
L. lactis Pmet-gfp	*L. lactis* wild-type strain MG1363, *llmg_pseudo10::Pmet-gfp*, Ery^r
L. lactis Δ*bcaP Pmet-gfp*	*L. lactis* MG1363 *bcaP* deletion mutant, *llmg_pseudo10::Pmet-gfp,* Ery^r
L. lactis Δ*met Pmet-gfp*	*L. lactis* MG1363 *met* deletion mutant, *llmg_pseudo10::Pmet-gfp,* Ery^r
L. lactis Δ*cmhR Pmet-gfp*	*L. lactis* MG1363 *cmhR* deletion mutant, *llmg_pseudo10::Pmet-gfp,* Ery^r
L. lactis Δ*rel Pmet-gfp*	*L. lactis* MG1363 *rel* deletion mutant, *llmg_pseudo10::Pmet-gfp,* Ery^r
L. lactis Δ*ccpA Pmet-gfp*	*L. lactis* MG1363 *ccpA* deletion mutant, *llmg_pseudo10::Pmet-gfp,* Ery^r
L. lactis Δ*codY Pmet-gfp*	*L. lactis* MG1363 *codY* deletion mutant, *llmg_pseudo10::Pmet-gfp*, Ery^r
L. lactis Δ*bcaP*Δ*brnQ Pmet-gfp*	*L. lactis* MG1363 *bcaP* and *brnQ* deletion mutant, *llmg_pseudo10::Pmet-gfp,* Ery^r
L. lactis Δ*met*Δ*bcaP*Δ*brnQ*	*L. lactis* MG1363 *met*, *bcaP* and *brnQ* deletion mutant
L. lactis Pmet(-RE)-gfp	*L. lactis* MG1363, *llmg_pseudo10::Pmet(-RE)-gfp. Pmet(-RE)* is a variant of the *met* promoter, lacking the full T-box riboswitch, Ery^r
L. lactis Δ*rel Pmet(-RE)-gfp*	*L. lactis* MG1363 *rel* deletion mutant, *llmg_pseudo10::Pmet(-RE)-gfp. Pmet(-RE)* is a variant of the *met* promoter, lacking the full T-box riboswitch, Ery^r
T-box mutant 1	*L. lactis* MG1363, *llmg_pseudo10::Pmet(mut1)-gfp.* Mutant 1 corresponds to G27T, G73T. Ery^r
T-box mutant 2	*L. lactis* MG1363, *llmg_pseudo10::Pmet(mut1)-gfp.* Mutant 2 corresponds to ΔUGGU. Ery^r
T-box mutant 3	*L. lactis* MG1363, *llmg_pseudo10::Pmet(mut1)-gfp.* Mutant 3 corresponds to C258U, G259U, U260C. Ery^r
T-box mutant 4	*L. lactis* MG1363, *llmg_pseudo10::Pmet(mut1)-gfp.* Mutant 4 corresponds to Δ306-365. Ery^r

Supplementary Table 3 | Number of generations in *L. lactis* colonies

	Colonies on CDM-agar plate with methionine 0.025 mM				Colonies on CDM-agar plate with methionine 10 mM	
	Phenotype				*Phenotype*	
	GFP + colonies		*GFP- colonies*		*GFP- colonies*	
number of coloy	number of cells	number of generations	number of cells	number of generations	number of cells	number of generations
1	2729	13.74	1515	12.89	1147	12.49
2	466	11.19	509	11.31	561	11.45
3	1379	12.75	1250	12.61	714	11.80
4	2624	13.68	1009	12.30	635	11.63
5	2492	13.61	1275	12.64	1158	12.50
6	945	12.21	513	11.32	723	11.82
7	590	11.53	1530	12.90	1053	12.36
8	1196	12.55	324	10.66	2178	13.41
9			726	11.83	3729	14.19
10			1675	13.03	1972	13.27
11					868	12.08
	mean ± SD	12.65 ± 0.98	mean ± SD	12.15 ± 0.82	mean ± SD	12.45 ± 0.85

Supplementary Table 4 | Regulation mechanism of the methionine transport in different phylogenetic clades

Phylogenetic clade	Regulation on methionine transport
Clostridia	S-box attenuation mechanism
B. subtilis group	S-box attenuation mechanism
B. cereus group	S-box attenuation mechanism
Lactobacilli	T-box attenuation mechanism
Staphylococci	S-box attenuation mechanism / T-box attenuation mechanism
Streptococci	Protein transcription factors (MtaR/MetR/CmbR)

Strategy in the regulation of methionine transporter in different phylogenetic clades, based on genomic studies. The S-box mechanism was lost in the Streptococcaceae and Lactobacillales lineages, and the role of regulation was assumed by the Met-T-box mechanism in lactobacilli and transcription factors in streptococci. Methionine auxotrophy has been reported in organisms belonging to the *Lactobacilli* and *Streptococci* clades.

Supplementary References

1. Bailey, T. L. & Gribskov, M. Combining evidence using p-values: Application to sequence homology searches. *Bioinformatics* **14,** 48-54 (1998).

2. Abreu-Goodger, C. & Merino, E. RibEx: A web server for locating riboswitches and other conserved bacterial regulatory elements. *Nucleic Acids Res.* **33,** 690-692 (2005).

3. Sperandio, B. *et al.* Three paralogous LysR-type transcriptional regulators control sulfur amino acid supply in *Streptococcus mutans*. *J. Bacteriol.* **192,** 3464-3473 (2010).

4. Liu, L. C., Grundy, F. J. & Henkin, T. M. Non-conserved residues in *Clostridium acetobutylicum* tRNAAla contribute to tRNA tuning for efficient antitermination of the *alaS* T Box riboswitch. *Life.* **5,** 1567-1582 (2015).

5. Zhang, J. *et al.* Specific Structural Elements of the T-Box Riboswitch Drive the Two-Step Binding of the tRNA Ligand. *Elife*. **7,** e39518 (2019).

6. Green, N. J., Grundy, F. J. & Henkin, T. M. The T box mechanism: tRNA as a regulatory molecule. *FEBS Letters.* **584,** 318-324 (2010).
