# Supplementary material for: A riboswitch gives rise to multi-generational phenotypic heterogeneity in an auxotrophic bacterium
Source: Nat Commun. 2020 Mar 5;11:1203. doi: 10.1038/s41467-020-15017-1 (PMC7058034; doi:10.1038/s41467-020-15017-1)
Supplement: Supplementary file 3 — Description of Additional Supplementary Files [file 41467_2020_15017_MOESM3_ESM.pdf]

Description of Additional Supplementary Files

File Name: Supplementary Movie 1

Description: L. lactis Pmet-gfp cells were grown on standard CDM containing methionine at a concentration of 0.27 mM, phenotypic heterogeneity is observed by time-lapse imaging.

File Name: Supplementary Movie 2

Description: L. lactis Pmet-gfp cells were grown on CDM containing low methionine concentrations (0.025 mM). The phenotypic heterogeneity is observed by time-lapse imaging.

File Name: Supplementary Movie 3

Description: L. lactis Pmet-gfp cells were grown on CDM containing high methionine concentrations (1 mM). The phenotypic heterogeneity disappears. Time-lapse imaging.

File Name: Supplementary Movie 4

Description: L. lactis Pmet-gfp cells were grown on CDM containing low methionine concentrations (0.025 mM). The phenotypic heterogeneity is present and few switching events are observed, cells with phenotype GFP- switch to phenotype GFP+. Time-lapse imaging.

File Name: Supplementary Movie 5

Description: L. lactis Δmet Pmet-gfp grown on CDM with methionine at a concentration of 1mM. This strain requires higher c oncentrations of methionine compared to the wild type. A concentration of methionine of 1 mM is low for this strain, therefore the met promoter is highly activated and no phenotypic heterogeneity is observed. Time-lapse imaging.

File Name: Supplementary Movie 6

Description: L. lactis Δmet Pmet-gfp grown on CDM with high methionine concentration (10mM). A concentration of methio nine of 10 mM provides enough amounts of methionine to this strain, therefore the expression of the met operon decreases (low fluorescence levels) and no phenotypic heterogeneity is observed. Time-lapse imaging.

File Name: Supplementary Movie 7

Description: L. lactis Pmet-gfp grown on CDM with methionine at a concentration of 0.025 mM and L-homocysteine (0.27 mM). Time -lapse imaging.

File Name: Supplementary Movie 8

Description: L. lactis Pmet-gfp grown on CDM with methionine at a concentration of 1 mM and L-homocysteine (0.27 mM). Time-lapse imaging.
